# Raising the redox potential in carboxyphenolate-based positive organic materials via cation substitution

**DOI:** 10.1038/s41467-018-06708-x

**Published:** 2018-10-23

**Authors:** Alia Jouhara, Nicolas Dupré, Anne-Claire Gaillot, Dominique Guyomard, Franck Dolhem, Philippe Poizot

**Affiliations:** 1 Institut des Matériaux Jean Rouxel (IMN), UMR CNRS 6502, Université de Nantes, 2 rue de la Houssinière, B.P. 32229, 44322 Nantes Cedex 3, France; 2 Laboratoire de Glycochimie, des Antimicrobiens et des Agroressources (LG2A), UMR CNRS 7378, Université de Picardie Jules Verne, 33 rue Saint-Leu 80039 Amiens Cedex, France; 3 Réseau sur le Stockage Électrochimique de l’Énergie (RS2E), FR CNRS 3459, France; 4 Institut Universitaire de France (IUF), 1 rue Descartes, 75231 Paris Cedex 05, France

## Abstract

Meeting the ever-growing demand for electrical storage devices requires both superior and “greener” battery technologies. Nearly 40 years after the discovery of conductive polymers, long cycling stability in lithium organic batteries has now been achieved. However, the synthesis of high-voltage lithiated organic cathode materials is rather challenging, so very few examples of all-organic lithium-ion cells currently exist. Herein, we present an inventive chemical approach leading to a significant increase of the redox potential of lithiated organic electrode materials. This is achieved by tuning the electronic effects in the redox-active organic skeleton thanks to the permanent presence of a spectator cation in the host structure exhibiting a high ionic potential (or electronegativity). Thus, substituting magnesium (2,5-dilithium-oxy)-terephthalate for lithium (2,5-dilithium-oxy)-terephthalate enables a voltage gain of nearly +800 mV. This compound being also able to act as negative electrode via the carboxylate functional groups, an all-organic symmetric lithium-ion cell exhibiting an output voltage of 2.5 V is demonstrated.

## Abstract (web summary)

Organic electrode materials could enable novelty chemistry required by the new generation of batteries. Here the authors show the synthesis and electrochemical performance of Mg(Li_2_)-*p*-DHT as a lithiated cathode material that cycles at 3.4 V due to the presence of a spectator cation in the host structure.

## Introduction

Rechargeable lithium-ion batteries (LIBs) used in portable electronic devices now number well over a billion units per year, and mid-term growth is expected. The pressure on the LIB market is further accentuated by the widespread global development of electric mobility which is naturally aligned with the emerging socio-technical transition. Being faced with such a high worldwide battery demand raises issues concerning resource availability and recyclability, which are further compounded by the challenges of providing the necessary technical requirements in terms of capacity, energy density, cyclability, safety and cost. This statement having naturally been anticipated^1^, numerous research efforts have been pursued in the quest for alternative chemistries. Among these, Li–S, Na-ion and metal–air systems have emerged as promising post Li-ion technologies^2–9^. After years of relative silence, the possibilities offered by organic electrode materials have also re-emerged in the energy storage community^10^—particularly following the development of the so-called organic radical batteries pioneered by the Nippon Electric Company (NEC) and Nishide and colleagues^11–15^—opening up many interesting opportunities such as design flexibility, lighter weight, lower cost and/or a tempered environmental burden^2,10^. Moreover, these materials offer the possibility of extending the conventional reversible cation uptake/release electrochemical mechanism (with n-type organic redox center) to include an anion-inserting process (with p-type organic redox center)^16–18^. They also unveil a whole new arena by making way for the design of organic electrochemical storage systems, including the development, in theory, of metal-free (molecular) ion batteries^16,19–23^. To date, a myriad of promising electroactive organic materials for application in non-aqueous (metallic) Li- or Na-based batteries have been investigated, leading to the publication of several reviews on the topic^9,15,24–29^. More recently, K-, Mg- and Al-inserting organic electrodes have also been investigated^30–34^. It should be mentioned that water-based electrolytes are also being considered with respect to promoting low-cost organic batteries^35–37^ due to their continued promising performance in redox flow technology, achieved by combining natural quinones and aqueous electrolyte media, notably thanks to the works of Aziz and authors^38–43^.

**Fig. 1:**
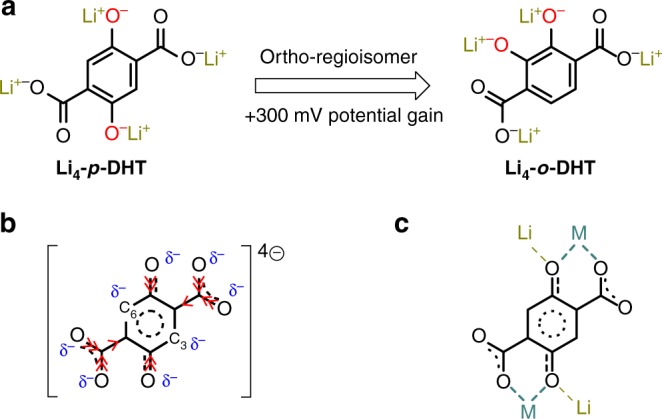
Electronic effects and potential tuning. **a** Formal potential increase when switching from para- to ortho-regioisomer in Li_4_DHT^51^. **b** Limiting structure (hybrid) of the tetranionic *p*-DHT ligand together with partial charges (in blue). The red arrows conventionally indicate the donor inductive effect (+I) related to both phenolate and carboxylate substituent groups. **c** Illustration of the chelating bonding mode between the tetranionic ligand and countercations based on crystallographic data reported for MOFs containing *p*-DHT as polytopic linker (i.e., Li^+^ and a spectator cation M^*n*+^); the nature of M−O bonds can affect the electronic distribution along the aromatic system

However, as recently underlined^23^, very few examples of all-organic Li-ion cells have been reported in the literature because of the inherent difficulty in designing efficient lithiated organic cathode materials, as opposed to their inorganic counterparts (e.g., LiCoO_2_, LiNi_1/3_Mn_1/3_Co_1/3_O_2_ or LiFePO_4_). Our group was the first to report an all-organic Li-ion cell based on renewable raw materials, by virtue of the amphoteric redox property of Li_4_C_6_O_6_ (Supplementary Fig. 1a) which led to the design of a cell exhibiting an ∼1 V output voltage^44,45^. This redox effectiveness of conjugated Li-enolate moieties (=C(OLi)−), as well as the synchronous discovery of the reversible reduction process associated with the carboxylate functional groups in terephthalate at very low potential (∼0.8 V vs. Li^+^/Li)^46^, prompted us to investigate the electrochemical behavior of dilithium (2,5-dilithium-oxy)-terephthalate (denoted Li_4_-*p*-DHT)^47^ as a promising dual-function electrode for a second generation of all-organic Li-ion cells, also entirely derived from renewable resources (Supplementary Fig. 1b). To date, Chen and colleagues^48^ has reported the best performance with this material, on condition that it is prepared in the form of nanosheets in order to reach its full theoretical capacity. As a result of the two-electron reactions formally occurring between Li_2_-*p*-DHT/Li_4_-*p*-DHT at ∼2.6 V on the positive electrode side and Li_4_-*p*-DHT/Li_6_-*p*-DHT at ∼0.8 V on the negative electrode side, respectively, this all-organic Li-ion cell exhibits an average operating voltage of 1.8 V and an energy density of about 130 Wh kg^−1^ together with a long cycling life (1000 cycles) when supported on graphene^49^. This fascinating organic skeleton urged us to further investigate the possibilities offered by its molecular engineering, especially with respect to increasing the formal redox potential of the Li-diphenolate ring. An obvious and attractive aspect of its organic chemistry is the multiplicity of possible chemical combinations at the molecular level giving rise to an incomparable tool for tuning several properties. This notably includes the redox potential, especially in conjugated systems when playing with both inductive and mesomeric effects^50–52^. Having decided to explore this avenue first, (i.e. focusing on the tuning of the organic part), the simplest strategy was to take advantage of the well-known phenomenon in molecular electrochemistry^50^ whereby a positive potential shift occurs when switching from the para- to the ortho-position in the quinone/hydroquinone moiety (Fig. 1a). After having made the appropriate adjustments to our initial synthesis approach, formerly developed for Li_4_-*p*-DHT, we succeeded in preparing the ortho-regioisomer, namely dilithium (2,3-dilithium-oxy)-terephthalate (Li_4_-*o*-DHT)^51^, and demonstrated an interesting voltage gain of +300 mV compared to Li_4_-*p*-DHT in the solid state, although this gain was not high enough to compete with LiFePO_4_, a common inorganic insertion electrode material used in commercial LIBs.

In the interest of surpassing this, we herein specifically report on the synthesis and electrochemical performance of magnesium (2,5-dilithium-oxy)-terephthalate (denoted Mg(Li_2_)-*p*-DHT) as a very promising lithiated positive material able to cycle at near 3.4 V vs. Li^+^/Li thanks to the presence of a magnesium cation in its chemical structure; a spectator ion exhibiting a high ionic potential value. Data on $M_{{\mathrm{2/}}n}^{n + }\left( {{{\mathrm{Li}}_2}}\right){\hbox{-}}p{ \hbox{-} \mathrm{DHT}}$ counterparts with M^*n*+^ = Li^+^, Ca^2+^ and Ba^2+^ are also provided to better understand the effect of the substitution chemistry on the redox properties of the Li-diphenolate ring in the tetranionic *p*-DHT ligand. As previously observed with the two regioisomers of Li_4_DHT^47,51^, we demonstrate that the reversible capacity is still limited to half of the theoretical value in our experimental conditions probably due to the high stability of the electrogenerated radical anion in the solid state. Last but not the least, in Mg(Li_2_)-*p*-DHT, being also reversibly electroactive in reduction through its carboxylate functional groups, the assembly of an all-organic symmetric Li-ion cell is shown exhibiting 2.5 V as output voltage.

## Results

### The concept

Inspired by the works of Lewandowski et al.^53^ pointing out the correlation between the perturbation of the electronic system of aromatic ligands (including 2-hydroxybenzoate, a parent structure of our ligand) and the position of the countercation in the periodic table, in this communication we demonstrate an equally intriguing approach for increasing the formal potential of redox-active conjugated Li-diphenolate ring in the *p*-DHT system by modifying the chemical nature of the spectator cation which formally compensates for the negatively charged carboxylate substituent groups. To better grasp this keen chemical stratagem based on the tuning of the p-electron density distribution in the aromatic skeleton, it is worth going over the electronic effects occurring in our tetranionic ligand. Figure 1b shows its limiting structure (hybrid) together with the inductive donor effect (+I) of both –O^−^ and –COO^−^ substituent groups. In short, one notes that the C(3) and C(6) carbon atoms (formally involved in the conjugated Li-diphenolate ring) display a higher electron density through mesomeric effects, while the four electron-donating substituent groups enrich the ring thereby making the diphenolate ring more reducing, which explains the resulting relatively low formal potential observed with Li_4_-*p*-DHT (∼2.6 V vs. Li^+^/Li). However, the presence of –COO^−^ groups remains essential in order to ensure a very low solubility of the organic material in the carbonate-based liquid electrolyte^9,15,47,51,54–57^. Figure 1c illustrates the chelating bonding mode between the organic tetraanion and the countercations (while maintaining Li^+^ on the phenolates), based on crystallographic data reported for metal organic frameworks (MOFs) using *p*-DHT as polytopic linker^58,59^. The nature and position of the metal has an influence on the M–O bonds, and this effect is transferred to the C–O bonds causing changes in the electronic distribution in the ligand. The stabilization (or destabilization) of the aromatic system thus depends on the choice of spectator cation M^*n*+^ and its relative ionic potential (or its electronegativity)^53^. Therefore, cations exhibiting a low ionic potential value should destabilize the distribution of p-electron density because they make weak bonds with oxygen atoms. Among the cations studied by Lewandowski et al.^53^ that stabilize the distribution of p-electron density (and that should increase the formal redox potential), magnesium was specifically selected for its abundance, low cost and low atomic weight. We also specifically decided to prepare the Ba(Li_2_)-*p*-DHT counterpart for comparison purposes since Ba^2+^ is also an alkaline-earth metal like Mg^2+^ but its ionic potential value is interestingly quite close to that of the Li alkali (Supplementary Table 1). Finally, Ca(Li_2_)-*p*-DHT was also integrated for making further comparisons.

### Preparation and characterizations of Mg(Li_2_)-*p*-DHT

**Fig. 2:**
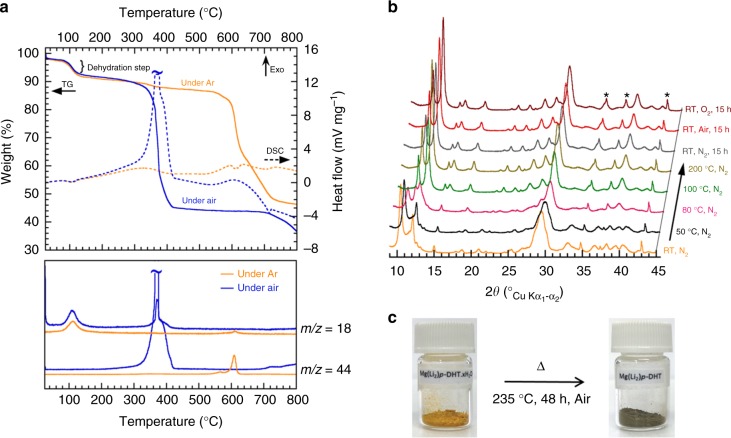
Thermal properties of Mg(Li_2_)-*p*-DHT·H_2_O. **a** Thermogravimetry (TG) and differential scanning calorimetry (DSC) traces of Mg(Li_2_)-*p*-DHT·H_2_O measured under argon or air flow at a heating rate of 5 °C min^−1^, combined with mass spectrometry (MS) data; *m/z* = 18 is ascribed to H_2_O, and 44 to CO_2_ evolutions, respectively. **b** TRXRPD patterns collected under nitrogen flow from RT to 200 °C. After an isotherm at 200 °C (16 h), the sample was cooled down to 20 °C and maintained under N_2_ for 15 h before changing the atmosphere for air, and then finally for pure O_2_ flow (* corresponds to the diffraction peak of the sample holder made of Al_2_O_3_). **c** The orange color of the pristine powder changed to a khaki color after the dehydration process at 235 °C for 48 h in air

A green approach was used to synthesize the Mg(Li_2_)-*p-*DHT. The first step consisted in neutralizing the two carboxylic functional groups of 2,5-dihydroxyterephthalic acid (H_4_-*p*-DHT) with a stoichiometric amount of magnesium hydroxide in water, under ambient atmosphere. Interestingly, after evaporation of the water under reduced pressure, this route promptly produced a batch of pure single crystals of Mg(H_2_)-*p*-DHT(H_2_O)_5_·H_2_O, as deduced by X-ray diffraction (XRD). The corresponding structure comprises five water molecules coordinated to the Mg cation bonding the *p*-DHT units, with one additional water molecule located in the inter-sheet spaces (Supplementary Fig. 2 and Supplementary Table 2). An equivalent structure was recently published by Henkelis et al.^60^, although a distinctly different synthesis approach was used. The as-produced organic salt was also characterized by thermal analysis, Fourier-transform infrared spectroscopy (FTIR) measurement and liquid/solid nuclear magnetic resonance (NMR) spectroscopy (Supplementary Fig. 3). The second step concerned the neutralization of the two phenolic groups in the presence of two equivalents of lithium hydroxide in water, under inert atmosphere. The heterogeneous yellow medium was then dried under vacuum at room temperature until an orange residue was obtained. The resulting orange powder was first characterized by FTIR and then by ^1^H/^13^C liquid NMR spectroscopy (Supplementary Fig. 4), which led to the conclusion that a hydrated form was obtained. Figure 2a shows both the thermogravimetry (TG) and differential scanning calorimeter (DSC) curves, measured either under argon or air, together with the corresponding mass spectrometry data. Unambiguously, the first weight loss of ~10.2% at 100 °C, associated with an endothermic phenomenon, corresponds to the release of one water molecule (as detected by mass spectrometry (MS)) giving rise to the following chemical formula: Mg(Li_2_)-*p*-DHT·H_2_O. The second large weight loss, occurring at 350 °C (under air) or at 600 °C (under argon), involves an obvious thermal decomposition of the compound. Note that Mg(Li_2_)-*p*-DHT exhibits a good thermal stability up to about 250 °C in air, which is an important factor in the safety of rechargeable batteries. The analysis of Mg(Li_2_)-*p*-DHT·H_2_O by temperature-resolved X-ray powder diffraction (TRXRPD) collected under N_2_ from room temperature up to 200 °C (Fig. 2b) revealed the occurrence of a phase transition process yielding the corresponding anhydrous phase as confirmed by FTIR measurement (Supplementary Fig. 4a).

**Fig. 3:**
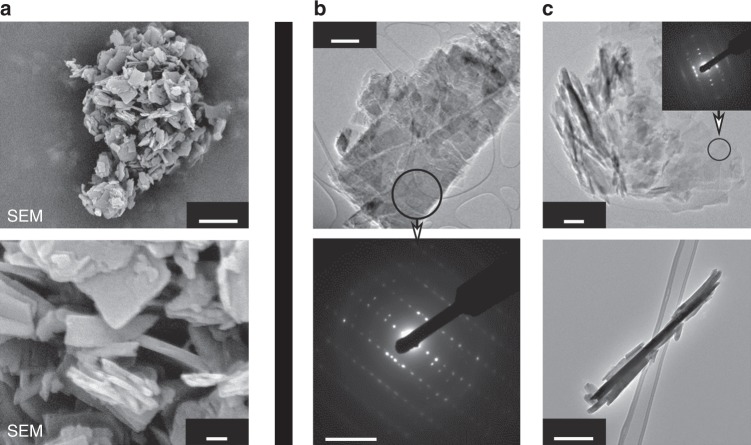
Characterizations by electron microscopy techniques. **a** Typical SEM images obtained by means of backscattered electrons of desolvated Mg(Li_2_)-*p*-DHT particles showing agglomerations of thin platelets. Scale bar represents 1 µm (up) and 100 nm (down), respectively. **b** TEM image of a large platelet together with the corresponding SAED pattern, recorded from the circled zone, revealing an orthogonal 2D cell of ~3.3 × 7.8 Å. Additional signal comes from slightly disoriented exfoliated layers. Scale bar represents 0.5 µm (up) and 5 nm^−1^ (down), respectively. **c** Additional view of the thin, partly exfoliated, creased or rolled particles, together with the same selected-area electron diffraction (SAED) pattern recorded on flat particles. Bottom: micron-long rolled particles forming a 40-nm tube. Scale bar represents 0.2 µm

After an isotherm step at 200 °C for 16 h, the sample (always inside the XRD high temperature chamber) was cooled down to 20 °C and subsequently exposed to various gas flows (N_2_, air, and finally pure O_2_ for 15 h each, Fig. 2b) in order to further study the chemical stability of the as-obtained anhydrous Mg(Li_2_)-*p*-DHT phase. Interestingly, no changes were observed—contrary to those noted for two regioisomers of Li_4_DHT^47,51^—thus indicating a good stability against O_2_ oxidation, and thereby supporting our strategy of replacing Li^+^ with Mg^2+^ in order to mitigate the donor inductive effects in the ring. The anhydrous Mg(Li_2_)-*p*-DHT phase was then produced on the gram-scale by thermal annealing of Mg(Li_2_)-*p*-DHT·H_2_O at 235 °C for 48 h under ambient air, producing a khaki powder (Fig. 2c). Scanning electron microscopy (SEM) revealed agglomerations of thin micron-sized platelets which were a few tenths of a nanometer thick (Fig. 3a).

Transmission electron microscopy (TEM) imaging confirmed the rectangular shape of unbroken individual particles, of which the largest measured 2 × 3 µm, and also piles of flaky particles mixed with others displaying a thin tubular morphology (~20 nm in diameter). The lamellar atomic structure of the materials is evidenced by its high tendency to exfoliate, crease or roll, as commonly observed for thin lamellar structures. The selected-area electron diffraction (SAED) pattern on the individual plate points to an orthogonal symmetry of the layers (~3.3 × 7.8 Å).

### Charge/discharge electrochemical testing of Mg(Li_2_)-*p*-DHT vs. Li

**Fig. 4:**
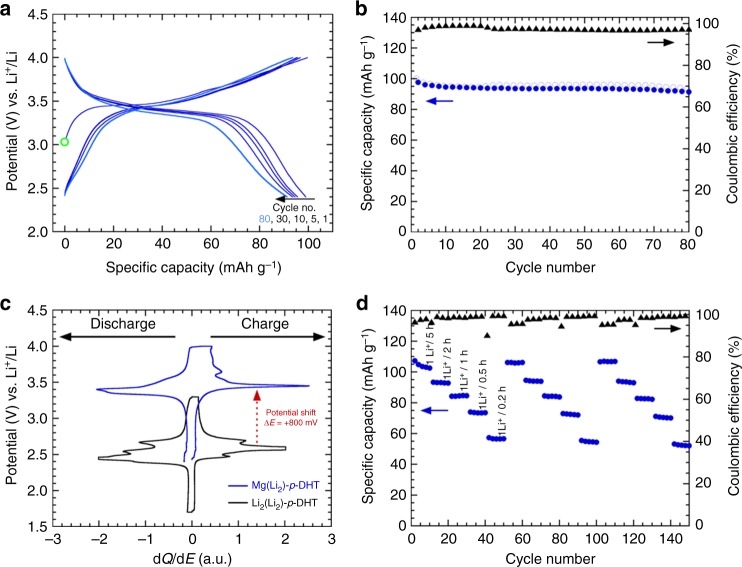
Charge/discharge electrochemical performance of Mg(Li_2_)-*p*-DHT electrode material vs. Li. **a** Potential vs. specific capacity curve (cycle nos. 1, 5, 10, 30 and 80) of a Li half-cell using Mg(Li_2_)-*p*-DHT as the active electrode material, mixed with 33 wt.% Ketjenblack EC-600JD (no binder) and galvanostatically cycled at a rate of 1 Li^+^/5 h (*I* = 23 mA g^−1^) LiPF_6_ 1 M in EC/DMC as the electrolyte. Note that the green circle indicates the starting open circuit potential (*E*_*I*=0_ ≈ 3.1 V vs. Li^+^/Li), which is notably higher than that of Li_4_-*p*-DHT (*E*_*I*=0_ ≈ 2.5 V vs. Li^+^/Li)^47^. **b** Corresponding capacity retention curve, together with the coulombic efficiency. **c** Superimposition of the potential vs. differential capacity curves (second cycle) for both Mg(Li_2_)-*p*-DHT (blue) and Li_4_-*p*-DHT (black) cycled vs. Li. The red arrow marks the positive potential shift. **d** Specific capacity vs. cycle number for current rate changes ranging from 1 Li^+^/5 h to 1 Li^+^/0.2 h per series of 10 cycles (repeated three times)

For the sake of comparison, the electrochemical investigations were performed vs. Li in Swagelok^®^-type cells, following a procedure similar to the one previously reported for the two Li_4_DHT regioisomers (i.e., simply mixing the active material with 33 wt.% conducting carbon without binder). We started by investigating this dual-function electrode material upon oxidation (i.e., operating as a positive electrode, Supplementary Fig. 1c) through the diphenolate electroactivity. Figure 4a shows the typical potential-specific capacity trace measured in galvanostatic mode, within the 2.4–4.0 V potential range, at a cycling rate of one Li^+^ exchanged per ligand in 5 h. As expected, Mg(Li_2_)-*p*-DHT behaves similarly to the two Li_4_DHT regioisomers by displaying an efficient reversible delithiation/lithiation process, which demonstrates the robustness of this organic chemical structure as well as its stability in carbonate-based liquid electrolytes. The most striking feature, nevertheless, is its impressively high average operating potential approaching 3.5 V vs. Li^+^/Li (Fig. 4a). This net potential gain of +800 mV compared to its Li_4_-*p*-DHT counterpart^47^ is better illustrated in Fig. 4c by simultaneously comparing the potential vs. differential capacity curves of Mg(Li_2_)-*p*-DHT and Li_4_-*p*-DHT. This outstanding result extends the series of considerations reported by Lewandowski et al.^53^ concerning the stabilizing effect of Mg^2+^ on the aromatic system to the domain of solid-state electrochemistry. Different cycling rates ranging from 1 Li^+^/5 h to 1 Li^+^/0.2 h, repeated three times, were also applied to another Mg(Li_2_)-*p*-DHT/Li half-cell in order to roughly assess the capability of the active material (Fig. 4d). It is worth noting that cycling with a stable restored capacity of almost 100 mAh g^−1^ coupled with good coulombic efficiency was obtained, even though the design of the composite electrode architecture was not optimized. Initial capacities were systematically recovered for each current rate change, indicating that the specific capacity loss at each current increase is purely due to kinetic limitations of the electrode; in other words, Mg(Li_2_)-*p*-DHT does not appear to be damaged at high cycling rates. In addition, no changing in the electrochemical behavior is noted upon cycling confirming the high stability of the Mg(Li_2_)-*p*-DHT electrode material (see cycle no. 1 and 80 in Fig. 4a). As expected, no exchange between Mg^2+^ cations chelated by carboxylate functional groups and Li^+^ from the surrounding electrolyte is observed. Indeed, if an ion exchange reaction occurred during the cycling, then the inductive effect due to the presence of Mg^2+^ should disappear and the measured redox potential should decrease towards that of Li_2_(Li_2_)-*p*-DHT (i.e., Li_4_-*p*-DHT). However, an apparent electrochemical reactivity restricted to roughly half of the expected 2-electron capacity (*Q*_th._ = 230 mAh g^−1^, Supplementary Fig. 1b, positive side) was measured by coulometry in the 2.4–4.0 V potential range, as was the case in our former studies on the two Li_4_DHT regioisomers^47,51^ which required further investigations (see below).

### Evidence of the charge perturbation by cationic substitution

**Fig. 5:**
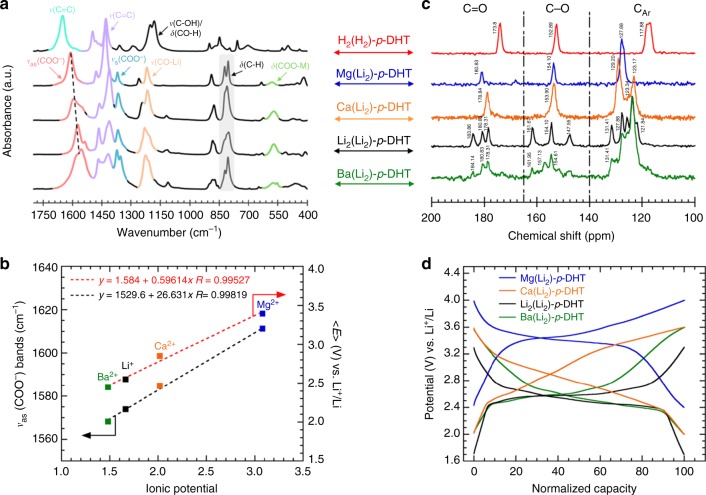
Charge perturbation in $M_{{\mathrm{2/}}n}^{n + }( {{\mathrm{Li}}_2}) {\hbox{-}}p{\hbox{-}}{\mathrm{{DHT}}}$ electrode materials (M^*n*+^ = Li^+^, Mg^2+^, Ca^2+^ and Ba^2+^. **a** Overlaid FTIR spectra of Ba(Li_2_)-*p*-DHT (green), Li_2_(Li_2_)-*p*-DHT (black), Ca(Li_2_)-*p*-DHT (orange) and Mg(Li_2_)-*p*-DHT (blue), including that of the acid form of the ligand, namely H_4_-*p*-DHT or H_2_(H_2_)-*p*-DHT (red), for comparison. **b** Correlation between both the antisymmetric vibrational modes of the carboxylate functional groups, *ν*_as_(COO^−^), and the average operating potential < *E* > (determined from differential capacity data) against the ionic potential of M^*n*+^. The dotted line is the linear fitting. **c** Overlaid ^13^C CP MAS-NMR spectra following the same order as in **a**. **d** Overlay of the as-obtained typical galvanostatic curve for each organic salt (2nd cycle) after normalization of the restored capacity (rate: 1 Li^+^/5 h, active material with 33 wt.% Ketjenblack EC-600JD; electrolyte: LiPF_6_ 1 M in EC/DMC)

Having been inspired by the works of Lewandowski et al.^53^ wherein Mg^2+^ was selected as a promising spectator cation, we decided to experimentally check the occurrence of this electronic charge perturbation within our particular tetranionic ligand by comparing the behavior of Mg(Li_2_)-*p*-DHT with Ba(Li_2_)-*p*-DHT, Ca(Li_2_)-*p*-DHT and Li_2_(Li_2_)-*p*-DHT, as indicated above. Note that the preparation and related characterizations of the two latter salts are detailed in the Supporting Information section. Among the various analytical tools used by Lewandowski et al.^53^ to assess the electronic charge perturbation in the ligand through the M–O bonds, FTIR spectroscopy proved to be the most suitable technique for probing solid-state electrode materials. Figure 5a displays the typical FTIR response of the three materials together, with the acid form spectrum for comparison; the typical assignment of infrared radiation (IR) vibration bands is listed in Supplementary Table 3.

For the four salts of the tetranionic *p*-DHT ligand, the deprotonation step is confirmed by the disappearance of the two characteristic bands of carboxylic acid groups located at 1648 cm^−1^ (ν_C=O_) and 1180 cm^−1^ (*ν*_C−O_/δ_C-O-H_), respectively. Indeed, the electron density is shared equally in the carboxylate anion between the two equivalent C−O bonds giving rise to an asymmetric mode (*ν*_as_) located in the 1550 and 1650 cm^−1^ regions, and a symmetric mode (*ν*_s_) observed between 1300 and 1420 cm^−1^. Of these two carboxylate modes, the position of the *ν*_as_(COO^−^) band is known to be sensitive to the electron density on the carboxylate carbon^61^; its shift to higher wavenumbers is correlated to an electron density decrease. Interestingly, the spectrum overlay shows a strong upshift of *ν*_as_(COO^−^) bands with Mg, unlike its Li, Ca, and Ba counterparts (Fig. 5a, pale red bands), indicating a stronger interaction with this cation and consequently a mitigation of the +I effect of the carboxylate anions towards the ring (stabilization of the aromatic system). This behavior is also well supported by the upshift of the aromatic bands (Fig. 5a, purple bands). In short, this FTIR study clearly confirms the mitigation effect on the electronic charge perturbation occurring in our tetranionic ligand depending on the chemical nature of the cation M, in accordance with the series of statements established by Lewandowski et al.^53^. As with these authors, one can distinctly observe a quasi-linear correlation when plotting *ν*_as_(COO^−^) against the ionic potential of M^*n*^^+^ (Fig. 5b).

These four organic materials were further probed in the solid state by magic-angle spinning (MAS) ^13^C NMR (Fig. 5c). Essentially, the spectra can be divided into three regions displaying characteristic resonances assigned to (***C***OO) (from 178 to184 ppm), to (***C***O) (from 147 to 162 ppm) and to (***C***_Ar_) carbons (121 to 132 ppm). As already observed for the IR spectra of Li_2_(Li_2_)-*p*-DHT and Ba(Li_2_)-*p*-DHT (Fig. 5a), their NMR counterparts also present striking similarities, displaying three resonances in the (***C***OO) and (***C***O) regions as well as a group of five overlapping resonances, all of which showing very similar chemical shifts. These complex NMR spectra particularly point to non-symmetric molecules with non-equivalent carbon atoms, as well as an identical overall configuration for these two compounds. Contrarily, the Ca(Li_2_)-*p*-DHT and Mg(Li_2_)-*p*-DHT ^13^C CP MAS-NMR spectra are more straightforward with only one resonance in each (***C***OO) and (***C***O) region and two resonances (strongly overlapping in the case of Mg(Li_2_)-*p*-DHT) in the ***C***_Ar_ region, which is indicative of a higher symmetry of the molecule, in agreement with the corresponding FTIR spectrum. The clear evolution observed in FTIR and electrochemical analyses from Ba(Li_2_)-*p*-DHT, Li_2_(Li_2_)-*p*-DHT and Ca(Li_2_)-*p*-DHT to Mg(Li_2_)-*p*-DHT can therefore also be monitored with NMR. We expected the effect of the electronegativity of the countercation on the ^13^C NMR chemical shift to be more visible on the most shielded carbons (i.e., those with the lowest chemical shifts), as is usually the case with the true electroactive center related to the redox-active phenolate functional group. At this point, it should be recalled that the hybrid shown in Fig. 1 exhibits a higher electron density for the C(3) and C(6) carbon atoms. Since Mg is more electronegative, its attractive effect can clearly be observed where it is easier to pull electrons out. Where Ba(Li_2_)-*p*-DHT, Li_2_(Li_2_)-*p*-DHT and Ca(Li_2_)-*p*-DHT are concerned, the most shielded aromatic carbons appear at 121.8, 123.3 ppm and 123.2 ppm (***C***_Ar_), in agreement with their electronegativity values of the same order of magnitude (Supplementary Table 1). The corresponding resonance appears at 127.9 ppm when Li^+^, Ba^2+^ or Ca^2+^ are replaced with Mg^2+^, consistent with its notably higher electronegativity value (χ = 1.29 based on the Allred–Rochow scale). Therefore, *δ*(***C***_Ar_) appears to be the appropriate NMR parameter here—although it appears to be somewhat less sensitive with respect to the variations of ν_C=O_ and ν_C=C_ in FTIR spectra—in order to monitor the influence of the M–O bonds on the distribution of p-electron density in the aromatic ring, by mitigating the donor inductive effects of the −COO^−^ group in particular. Indeed, although the larger difference in electronegativity values and electrochemical potentials between Ba(Li_2_)-*p*-DHT, Li_2_(Li_2_)-*p*-DHT and Ca(Li_2_)-*p*-DHT on one hand and Mg(Li_2_)-*p*-DHT) on the other hand translates in a significant difference in terms of chemical shifts, the smaller difference observed between cases of Ba(Li_2_)-*p*-DHT, Li_2_(Li_2_)-*p*-DHT and Ca(Li_2_)-*p*-DHT is barely observed in terms of their respective chemical shift. Finally, we decided to go a step beyond the works of Lewandowski et al.^53^ by also trying to correlate the average operating potential <*E*> (Fig. 5d) of each salt to the ionic potential of M^*n*+^, and managed to obtain a very good linear fit (Fig. 5b). To our knowledge, this is the first time that such a correlation is being reported.

### Ex situ and in situ characterizations of the one-electron limited electrochemical process

**Fig. 6:**
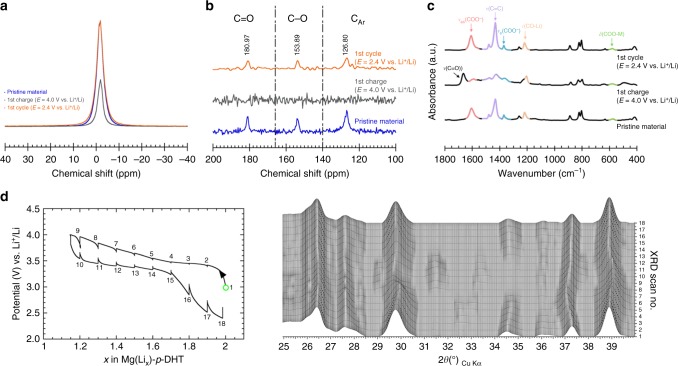
Supporting data for one-electron limited electrode process. **a** Ex situ ^7^Li MAS-NMR spectra. **b** Corresponding ex situ ^13^C CP MAS-NMR spectra using micrometric-sized Pt particles in place of carbon black conducting additive. **c** FTIR data for Mg(Li_2_)-*p*-DHT-based electrodes containing 33 wt.% Ketjenblack EC-600JD before cycling (blue), after a first charge up to 4 V vs. Li^+^/Li (gray) and one full cycle (orange), within the 2.4–4.0 V potential range (rate: 1 Li^+^/5 h). **d** Potential-composition profile for a Mg(Li_2_)-*p*-DHT/Li in situ XRD electrochemical cell^63^ together with the corresponding collected XRD patterns over the 10–40° 2*θ* range. The cell was cycled in the 2.4–4.0 V potential range using an intermittent galvanostatic mode. More specifically, a current of 11.2 mA per g of Mg(Li_2_)-*p*-DHT (rate: 1 Li^+^/10 h) was applied for a period of 2 h, separated by resting periods of 1 h during which the XRD patterns were collected (electrolyte: LiPF_6_ 1 M in EC/DMC)

As stated above, in our experimental conditions, all coulometry measurements performed on $M_{{\mathrm{2/}}n}^{n + }( {{\mathrm{Li}}_2}) {\hbox{-}} - p{\hbox{-}}{\mathrm{{DHT}}}$ electrode materials (M = Li^+^, Mg^2+^, Ca^2+^ and Ba^2+^) systematically indicated a peculiar reversible process restricted to a one-electron reaction, although a very good performance was shown upon cycling (see ref. ^53^ for M^*n*+^ = Li^+^, Fig. 5 for M^*n*+^ = Mg^2+^, Supplementary Fig. 5 for M^*n*+^ = Ba^2+^, Supplementary Fig. 6 for M^*n*+^ = Ca^2+^). Interestingly, Chen and colleagues^48,49^ overcame this experimental factor with Li_2_(Li_2_)-*p*-DHT by synthesizing the material in the form of nanosheets. It appeared to be necessary, however, to confirm our results by employing further analytical techniques. First, ^7^Li MAS-NMR was chosen as a powerful quantitative method for monitoring the amount of lithium ions involved in the redox and electrochemical reactions, since the integrated intensity of the acquired signal is directly proportional to the amount of nuclei under observation in the sample^62^. In the present case, we opted for a normalization of integrated intensities for the sake of simplicity, using 2 and 4 values for the pristine Mg(Li_2_)-*p*-DHT (Fig. 6a) and Li_2_(Li_2_)-*p*-DHT (Supplementary Fig. 6a) composite electrodes, respectively. Integrated intensities for the Li_2_(Li_2_)-*p*-DHT electrode after the 1st charge (oxidation), and after one cycle (subsequent reduction), were 3.1 and 4.1, respectively, supporting the fact that only one Li^+^/ring reacts. However, caution should be exercised with respect to this overall value because some particles may not be reacting. In the hypothesis of an incomplete electrical percolation within the composite electrode leading to the disconnection of exactly half of the active particles, the signature of the pristine (unreacted) Li_2_(Li_2_)-*p*-DHT material would certainly be detected by ^13^C NMR after oxidation. However, there was neither a trace of the pristine compound detected at the end of oxidation nor a trace of the oxidized compound observed after one charge–discharge cycle (Supplementary Fig. 6b). Similarly, for Mg(Li_2_)-*p*-DHT (Fig. 6a), integrated intensities after one oxidation and after one cycle were 1.2 and 2.2, respectively, again supporting the occurrence of a one-electron reaction. In addition to this, ^13^C CP MAS-NMR experiments were also performed with Mg(Li_2_)-*p*-DHT, although the low signal to noise ratio did not permit the acquisition of exploitable data due to the presence of conductive carbon within the electrode. With the purpose of facilitating the observation of the natural abundance ^13^C signal, without it being hindered by the ^13^C NMR signal stemming from the carbon additive (Supplementary Fig. 6b), we developed a novel electrode formulation wherein the carbon additive was replaced with Pt micrometric-sized particles to ensure electronic conductivity within the composite electrode. To the best of our knowledge, an electrode formulation of this type has never been used before, and has proven to be invaluable for monitoring the ^13^C CP MAS-NMR evolution upon cycling. Spectra corresponding to Mg(Li_2_)-*p*-DHT after one oxidation and one cycle (Fig. 6b) clearly indicate that the pristine material is recovered after one full electrochemical cycle. The absence of signal for the oxidized material could be assigned to the formation of the radical form of the active organic material (Mg(Li)-*p*-DHT^•^), thereby inducing an extremely short NMR signal relaxation time, and thus leading to an extremely broad resonance which is unobservable under fast MAS, even in low field conditions. Moreover, the formation of a radical form of the material supports the one-electron reaction. Therefore, the combined ^7^Li and ^13^C NMR results unambiguously indicate that only one Li^+^/ring is reversibly involved in the overall electrochemical reaction. The slight variation in terms of the integrated intensity observed for the cycled samples could either be ascribed to Li in the passivation layer on the surface of the electrode or to remaining unreacted LiPF_6_ salt from the electrolyte. Additionally, the reversibility of the electrochemical reaction was assessed by means of ex situ FTIR measurements during the first cycle (Fig. 6c). Upon oxidation, FTIR data revealed a reversible process with the emergence of a new band at ~1700 cm^−1^, which can be assigned to the carbon–oxygen stretching bands of the quinone or semiquinone radical groups. However, the slight alteration of *ν*_as_(C−O^−^) bands at ~1250 cm^−1^ seems to confirm the formation of a semiquinone radical since no bands are expected to appear in this region if the quinone form of the ligand is produced^48^. Finally, in situ XRD measurements (Fig. 6d) clearly emphasize the reversibility of the electrode reaction in the solid state. The relatively sharp Bragg peak at 39.3° broadens and shifts progressively towards higher angles at 39° upon the one-electron oxidation. The subsequent reduction sees the same Bragg peak sharpening and returning to its initial position.

By way of summary, coulometric measurements, combined ^7^Li and ^13^C NMR data, as well as FTIR spectroscopy, confirm the occurrence of a one-electron reaction per ring in all three representative salts, thus illustrating the fact that the electrochemical reaction seems to occur reversibly between $M_{{\mathrm{2/}}n}^{n + }( {{\mathrm{Li}}_2}) {\hbox{-}} p{\hbox{-}}{\mathrm{{DHT}}}$ and $M_{{\mathrm{2/}}n}^{n + }( {{\mathrm{Li}}}){\hbox{-}} p {\hbox{-}}{\mathrm{{DHT}}}$^•^ (radical) without reaching full oxidation into the quinone form (Supplementary Fig. 1c, positive side). Interestingly, a parallel can be drawn here with the p-type poly(vinylphenothiazine) electrode material reported by Esser and colleagues^17^, which enables a specific capacity that is also half of the theoretical one. In their study, the reversible reaction occurs between the radical cation and the cationic states, without restoring the original neutral state of the pristine polymer; a symmetrical situation occurs in our n-type electroactive system. The authors explained this incomplete electrode reaction as being the result of a peculiar stabilizing effect due to intra-chain or inter-molecular π–π interactions between phenothiazine units. This situation may well also apply to our lamellar organic scaffold. Chen and colleagues^48,49^ succeeded in obtaining the full theoretical capacity with Li_2_(Li_2_)-*p*-DHT, perhaps because the stacking height of a pile is significantly decreased in nanosheets and thereby alters the stabilizing π–π interactions.

**Fig. 7:**
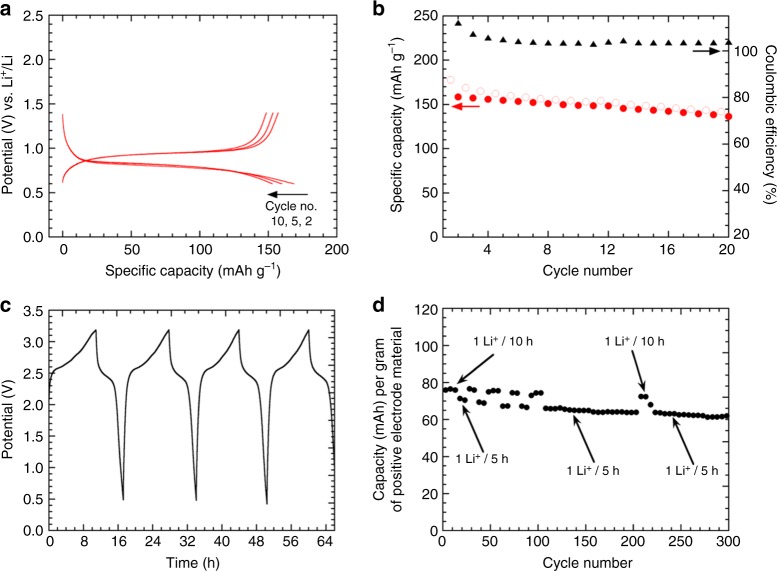
All-organic symmetric Li-ion cell based on Mg(Li_2_)-*p*-DHT. **a** Potential vs. specific capacity curve (cycle nos. 2, 5 and 10) of a Li half-cell using Mg(Li_2_)-*p*-DHT as the active electrode material, mixed with 33 wt.% GM15 (no binder) and galvanostatically cycled at a rate of 1 Li^+^/5 h (*I* = 11.5 mA g^−1^) in the 0.6–1.4 V potential range using LiPF_6_ 1 M in EC/DMC as the electrolyte. **b** Corresponding capacity retention curve. **c** Charge–discharge profile of the symmetric Li-ion cell made of Mg(Li_2_)-*p*-DHT with a ratio of 1:1 showing an output voltage of 2.5 V (*I* = 11.5 mA g^−1^ based on the weight of Mg(Li_2_)-*p*-DHT contained in the positive electrode). **d** Specific capacity vs. cycle number for current rate changes ranging from 1 Li^+^/10 h to 1 Li^+^/5 h

Based on this encouraging outcome, it seemed relevant to conclude this study by assembling an all-organic symmetric Li-ion cell, since Mg(Li_2_)-*p*-DHT was also expected to function as negative electrode material through the dicarboxylate functional groups (Supplementary Fig. 1b, c). Figure 7a summarizes the stable electrochemical profile obtained from the second cycle at low potential in a Li half-cell due to the electrochemical activity of the carboxylate functional groups. The first discharge curve exhibits the common extra-capacity observed with carboxylate-based organic electrodes^29,48,54–56^ together with an average operating potential of ~0.8 V vs. Li^+^/Li similar to the one observed with the dilithium terephthalate parent compound^46^. Therefore, the chemical nature of the spectator cation (M^*n*+^) seems to particularly influence the formal potential of the diphenolate ring and not that of the attached redox-active carboxylate moieties. Interestingly, several studies have also reported this stable operating potential with various alkali and alkaline-earth metal salts of terephthalate during the reversible lithiation/delithiation electrochemical process^64–66^. Without optimization, a reversible insertion/uptake of 1.35 Li^+^ per formula unit can be achieved leading to specific capacity value of 157 mAh g^−1^. After 20 cycles (Fig. 7b), this composite electrode was transferred to a 3-electrode cell and used as negative electrode. The positive electrode consisted of a fresh Mg(Li_2_)-*p*-DHT-based composite (mixed with 25 wt.% Ketjenblack EC-600JD) acting as the positive electrode; Li was used as the reference electrode. Figure 7c shows the corresponding cycling curve exhibiting an interesting average output voltage of 2.5 V consecutive to the mitigation of the inductive effects in the organic scaffold, via Mg^2+^ for the positive side. For comparison, Chen and colleagues^48,49^ reported a value of 1.8 V for the symmetric Li-ion cell based on Li_2_(Li_2_)-*p*-DHT. Finally, Fig. 7d exhibits the corresponding capacity retention curve calculated on the basis of the mass of the active material contained in the positive electrode and for different cycling rates. After 300 cycles, 82% of the initial capacity is still obtained.

## Discussion

In this study, we have demonstrated that an outstanding potential increase can be achieved in solid carboxyphenolate salts by mitigating donor inductive effects in the organic core thanks to the presence of spectator cations within the host structure exhibiting high ionic potential (or electronegativity). Thus, substituting magnesium (2,5-dilithium-oxy)-terephthalate for lithium (2,5-dilithium-oxy)-terephthalate enables a net voltage gain of nearly +800 mV during the reversible delithiation/lithiation electrode process—the formal redox-active organic skeleton remaining unchanged. It must be emphasized that this phenomenon, which involves subtle electrostatic effects within the chemical scaffold, should not be confused with the common potential shift related to a complexation reaction between a redox-active organic anion and a mobile cation coming from the electrolyte; the potential shift being proportional to the strength of the anion-cation pairing^67^. To a certain extent this finding is akin to the renowned “inductive effect” described by Goodenough and coworkers^68,69^ regarding the tuning of the Fe^3+^/Fe^2+^ redox potential in NASICON-type structures of the general formula A_*x*_Fe_2_(XO_4_)_3_, depending on the chemical nature of the polyanion XO_4_^*n−*^. The inductive effect of the polyanion, which depends on the electronegativity of X, weakens or strengthens the covalency of the Fe−O bonds and thereby changes the operating potential^70^. In our case the situation is simply reversed, since the redox center is the anion and not the cation. Based on several characterization techniques, this study was also the occasion to underline (i) the good electrochemical performance of Mg(Li_2_)-*p*-DHT for use as a positive lithiated electrode material, giving rise to a stable capacity retention of ~100  Ah g^−1^ over several dozen cycles (340 Wh kg^−1^), (ii) a clear limitation at half of the theoretical capacity (one-electron reaction per ring), which is probably due to stronger stabilizing effects in the radical anion state, and (iii) the demonstration of an all-organic symmetric Li-ion cell showing 2.5 V as output voltage giving rise to an energy density of about 89 Wh per kg of active electrode materials. Ongoing investigations using computational methods are in progress to better understand these distinctive results.

## Methods

### Synthesis

The synthetic protocols of the series of compounds reported in this study, as well as the common characterization techniques used (e.g., thermal and multi-elemental analyses, FTIR, liquid NMR, XRD, SEM-TEM), are thoroughly explained in supplementary information section.

### Electrochemistry

For the sake of comparison, electrochemical investigations were performed in Swagelok^®^-type cells, according to a procedure similar to the one previously reported for the two regioisomers Li_4_DHT^47,51^, using a Li metal disc as the negative electrode and a glassfiber separator soaked with LiPF_6_ 1 M in ethylene carbonate/dimethyl carbonate (EC/DMC; 1:1 vol./vol.) as the electrolyte (*V* ~600 µL). The composite positive electrode was prepared without a binder in an argon-filled glovebox by grinding the powder of the active organic materials and carbon additive with a mortar and pestle; the carbon content of the final electrode being 33% in mass. For the symmetric cell measurements, a Swagelok^®^-type 3-electrode cell was employed using Li metal as the reference electrode. Prior to assembling the symmetric cell, the negative electrode material was first pre-cycled in the 1.4–0.6 V vs. Li^+^/Li potential range, until having achieved a satisfactory stability upon cycling coupled with a coulombic efficiency value close to 100% (i.e., 20 cycles, rate: 1 Li^+^/10 h, *I* = 11.5 mA g^−1^, Fig. 7a), as is reported in the literature^48^. The negative electrode composition was 63 wt.% Mg(Li_2_)-*p*-DHT, 32 wt.% graphite nanoplatelets (GM15) and 5 wt.% PTFE, whereas the positive electrode composition was 70 wt.% Mg(Li_2_)-*p*-DHT, 25 wt.% Ketjenblack EC-600JD and 5 wt.% PTFE. Each composite electrode was pressed at 1 ton cm^−2^ on a stainless steel (AISI 316L) grid current collector. An excess of 30% in capacity was used for the negative electrode to balance the 3-electrode cell (Fig. 7b). The positive side was controlled between 2.4 and 4 V vs. Li^+^/Li, whereas the negative side was controlled between 0.6 and 1.4 V vs. Li^+^/Li. All cells were cycled in galvanostatic mode using a MPG-2 multi-channel system (Bio-Logic SAS, Claix, France).

### MAS-NMR investigations

The solid-state MAS-NMR spectra of all studied compounds were acquired on a Bruker Avance 500 spectrometer (*B*_0_ = 11.8 T) using R.F. pulses at Larmor frequencies of 500, 194.0 and 125.7 MHz for ^1^H, ^7^Li and ^13^C, respectively. The samples were packed in 2.5 mm diameter zirconia rotors for ^1^H→^13^C CP MAS-NMR and spun at 10 kHz. ^7^Li NMR was performed at a spinning rate of 25 kHz with a 90° pulse length of 1.4 ms. Long recycle times of 60 s were used in order to avoid saturation of the signal and to measure quantitative results. For ex situ ^7^Li/^13^C MAS-NMR (as well as for ex situ FTIR), the positive composite electrode was prepared by mixing a powder of Mg(Li_2_)-*p*-DHT or Li_2_(Li_2_)-*p*-DHT (66 wt.%) with carbon black (33 wt.%) (typically, Ketjenblack EC-600JD from AkzoNobel) and cycled in galvanostatic mode at a typical rate of one lithium ion exchanged per ring in 5 h. For ex situ ^13^C CP MAS-NMR experiments performed on the Mg(Li_2_)-*p*-DHT electrodes, the cathode was prepared by grinding a powder of Mg(Li_2_)-*p*-DHT (15 wt.%) and platinum (85 wt.%) with a mortar and pestle. The platinum powder (amorphous) was purchased from Alfa Aesar (APS < 3 µm, 99.9%, tap density 0.6–2.0 g cm^−3^). The completed electrode was then cycled in galvanostatic mode at a rate of one lithium ion exchanged per ring in 50 h. After cycling, the Swagelok-type cells were disassembled in glovebox. For each cell, the positive electrode was collected and rinsed repeatedly with DMC and finally dried under vacuum at 60 °C for 5 h. The obtained samples (pristine, after one charge and one discharge) were then analyzed by ^7^Li MAS-NMR, ^13^C MAS-NMR, ^13^C CP MAS-NMR and FTIR spectroscopies.

## Electronic supplementary material

Supplementary Information

## Data Availability

The data supporting the findings of this study are available from the corresponding author on reasonable request.
