# Supplementary material for: Raising the redox potential in carboxyphenolate-based positive organic materials via cation substitution
Source: Nat Commun. 2018 Oct 23;9:4401. doi: 10.1038/s41467-018-06708-x (PMC6199296; doi:10.1038/s41467-018-06708-x)
Supplement: Supplementary file 1 — Supplementary Information [file 41467_2018_6708_MOESM1_ESM.docx]

Supplementary Information

Raising the Redox Potential in Carboxyphenolate-Based Positive Organic Materials via Cation Substitution

Jouhara et al.

**Supplementary Figures**





Supplementary Figure 1 | All-organic symmetric lithium-ion cells. a Using the tetralithium salt of tetrahydroxybenzoquinone (Li_4_C_6_O_6_ or Li_4_THQ)^1^. b Using the dilithium (2,5-dilithium-oxy)-terephthalate (Li_4_-*p*-DHT or “Li_2_(Li_2_)-*p*-DHT” in the present study)^2,3^. c Transposition to the magnesium (2,5-dilithium-oxy)-terephthalate, Mg(Li_2_)-*p*-DHT

**Supplementary Figure 2 | Comparison between single crystal XRD and powder XRD of Mg(H_2_)-*p*-DHT(H_2_O)_5_⋅H_2_O. a** Structure of Mg(H_2_)-*p*-DHT(H_2_O)_5_⋅H_2_O. **b** X-Ray powder diffraction pattern (final Rietveld refinement) of Mg(H_2_)-*p*-DHT(H_2_O)_5_⋅H_2_O measured 295 K

**Supplementary Figure 3 |** **Characterizations by liquid and solid NMR** **of Mg(H_2_)-*p*-DHT(H_2_O)_5_⋅H_2_O.** **a** Typical FTIR spectrum (KBr pellet). **b** TG/DSC traces measured under argon at a heating rate of 5°C min^−1^. **c** ^1^H liquid NMR spectrum measured in D_2_O. **d** ^13^C liquid NMR spectrum measured in D_2_O. **e** ^13^C CP MAS-NMR spectrum

Supplementary Figure 4 | Characterizations of Mg(Li_2_)-*p*-DHT. a Typical FTIR spectrum of the as-prepared Mg(Li_2_)-*p*-DHT (orange) showing the presence of water (hydrated phase) and after dehydration step (blue) at 235°C for 48 h under ambient air (KBr pellet). b ^1^H liquid NMR spectrum of the as-prepared Mg(Li_2_)-*p*-DHT measured in D_2_O showing high water content in agreement with the formation of a hydrated phase. b ^13^C liquid NMR spectrum of the as-prepared Mg(Li_2_)-*p*-DHT measured in D_2_O

Supplementary Figure 5 | Charge/discharge electrochemical performance of Ba(Li_2_)-*p*-DHT electrode material vs. Li. a Potential vs. specific capacity curve (cycle no. 1, 5, 10) of a Li half-cell using Ba(Li_2_)-*p*-DHT as the active electrode material mixed with 33 wt.% of Ketjenblack EC-600JD and galvanostatically cycled in EC:DMC / LiPF_6_ 1 M at a rate of 1 Li^+^/10 h rate (*I* = 7.8 mA per g of Ba(Li_2_)-*p*-DHT). b Corresponding capacity retention curves

Supplementary Figure 6 | Charge/discharge electrochemical performance of Ca(Li_2_)-*p*-DHT electrode material vs. Li. a Potential *vs.* specific capacity curve (cycle no. 1, 5, 10) of a Li half-cell using Ca(Li_2_)-*p*-DHT as the active electrode material mixed with 33 wt.% of Ketjenblack EC-600JD and galvanostatically cycled in EC:DMC / LiPF_6_ 1 M at a rate of 1 Li^+^/10 h rate (*I* = 7.8 mA per g of Ca(Li_2_)-*p*-DHT). b Corresponding capacity retention curves

**Supplementary Figure 7 | NMR study of lithiation / delithiation process of Li_2_(Li_2_)-*p*-DHT.** **a** *Ex situ* ^7^Li MAS-NMR spectra for Li_2_(Li_2_)-*p*-DHT-based electrodes containing 33 wt.% Ketjenblack EC-600JD before cycling (blue), after a first charge up to 3.5 V vs. Li^+^/Li (grey) and one full cycle (orange) within the 2.0-3.5 V vs. Li^+^/Li potential range (rate: 1 Li^+^/5 h). **b** ^13^C CP MAS-NMR spectra of Li_2_(Li_2_)-*p*-DHT electrodes before cycling (blue), at the end of the first oxidation up to *E* = 3.5 V vs. Li^+^/Li, in grey), and after one cycle (orange)

**Supplementary Tables**

**Supplementary Table 1 |** χ_AR_(M) represents the Allred-Rochow electronegativity value for M; Δχ_AR_ represents the electronegativity variation related to the M−O bond using χ_AR_(O) = 3.5; *r*_ion_ is the ionic radius of M (in Å) whereas [AOCN] corresponds to the average observed coordination numbers according to Brown’s review article^5^

| M^(n+)^···O  coordination bond | χAR(M) | ΔχAR | *r*_ion_ (Å) & [AOCN] | Ionic potential  (n / *r*)_ion_ | Average potential <*E*> (V) vs. Li^+^/Li |
| --- | --- | --- | --- | --- | --- |
| Mg^(2+)^···O | 1.29 | 2.21 | 0.65 [5.98] | 3.076 | 3.4 |
| Li^(+)^···O | 0.97 | 2.53 | 0.6 [5.3] | 1.666 | 2.55 |
| Ca^(2+)^···O | 1.04 | 2.49 | 0.99 [7.31] | 2.020 | 2.90 |
| Ba^(2+)^···O | 0.89 | 2.61 | 1.35 [10.24] | 1.481 | 2.45 |

**Supplementary Table 2 |** Comparison between single crystal and powder data for Mg(H_2_)-*p*-DHT(H_2_O)_5_⋅H_2_O measured 295 K. Note that such crystallographic data are similar to those recently reported by Henkelis et *al*.^4^

|  | Single crystal | Powder |
| --- | --- | --- |
| Temperature (K) | 295 | 295 |
| *M* (g mol^-1^) | 328.52 | 328.52 |
| Crystal symmetry | Monoclinic | Monoclinic |
| Space group | *P*2_1_/*n* | *P*2_1_/*n* |
| *a* (Å) | 10.1941(7) | 10.1898(4) |
| *b* (Å) | 6.6984(7) | 6.7003(2) |
| *c* (Å) | 20.7621(19) | 20.7524(7) |
| *β* (°) | 103.624(8) | 103.6159(18) |
| *V* (Å^3^) | 1377.8(2) | 1377.05(8) |
| Z, Z’ | 4, 1 | 4, 1 |

**Supplementary Table 3 |** Typical assignments of IR vibration bands reported in Figure 5a

**
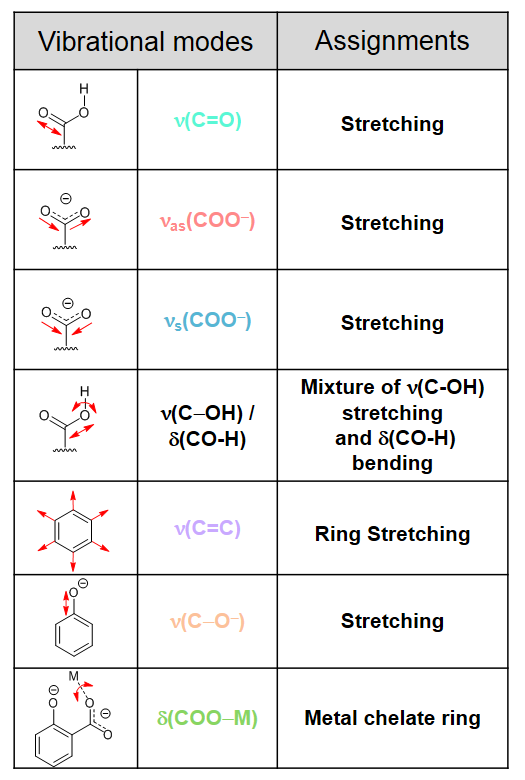
**

**Supplementary Methods**

**General analytical techniques.** FTIR measurements were recorded using KBr pellets in the range 4000 − 400 cm^−1^ on a FTIR Bruker Vertex 70. Air-free pellets were prepared in an argon glove box by mixing the sample with spectroscopic-grade potassium bromide at 1 wt.%. ^1^H and ^13^C liquid NMR spectra were recorded on a Bruker AVANCE III 400 MHz. Chemical shifts (δ) are given in ppm relative to TMS. NMR solvents used were purchased from Aldrich Company (purity higher than 99.5%). Elemental analyses were carried out using a Thermo Scientific Flash 2000 CHN analyzer. Inductively Coupled Plasma-Optical Emission Spectrometry (ICP-OES) obtained quantitative analyses of lithium/sodium/magnesium thanks to a Thermo Fischer Scientific iCAP7600 analyzer. The differential scanning calorimetry (TG-DSC) experiments were carried out under argon with a SENSYSevo instrument from Setaram using a heating rate of 5°C⋅min^−1^ up to 800°C. The thermogravimetry (TG)-Mass spectrometry (MS) coupled experiments were carried out with a STA449F3 Jupiter and QMS403C Aëolos instruments from NETZSCH under argon at a heating rate of 5°C⋅min^−1^ up to 800°C.

**X-ray diffraction techniques.** Temperature-resolved X-ray powder diffraction (TRXRPD) patterns collected with a Bruker D8 Advance powder diffractometer equipped with an Anton Parr XRK900 high temperature chamber. Data were collected in the Bragg-Brentano geometry with a Cu-anode X-ray source operated at 40 kV and 40 mA. The Cu K_β_ radiation was filtered by means of a Ni foil. The experiments were carried out under nitrogen flow in the 5-45° 2*θ* range with a step of 0.016°, an acquisition time of 1.2 s per step and a heating rate of 0.1 °C⋅s^−1^ under N_2_ from 20 to 200°C.

For single crystal X-ray diffraction technique, data collections were carried out at *T* = 173 K with a Bruker-Nonius Kappa CCD diffractometer using graphite monochromated Mo K_α_ radiation and equipped with an Oxford Cryosystems N2 cryostream.

*In situ* X-ray diffraction powder patterns were recorded using a PANanalytical X’Pert Pro diffractometer operated in Bragg-Brentano reflection geometry with a Long Line Focus Cu-anode X-ray source, and a X’Celerator RTMS detector. Data were collected in the 10-40° 2*θ* range with a step of 0.017° and an acquisition time of 1.9 s per step. Measurements were performed with a the Multi Purpose Sample Stage (MPSS) able to accommodate the *in situ* Swagelok-type cell previously described in the literature^6^. Note that the common beryllium window was removed because Be was unstable in the tested electrochemical conditions. Therefore, we replaced the beryllium window by a thin Kapton foil (X-ray film polyimide Kapton®, PANalytical B-V); thickness: 10 µm, diameter: 6.4 mm (see below). A Li metal disk was used as negative electrode whereas the positive electrode composition was 70 wt.% of Mg(Li_2_)-*p*-DHT, 25 wt.% Ketjenblack EC-600JD, and 5 wt.% PTFE. This mixture was pressed at 1 ton on a stainless steel (AISI 316L) grid current collector.


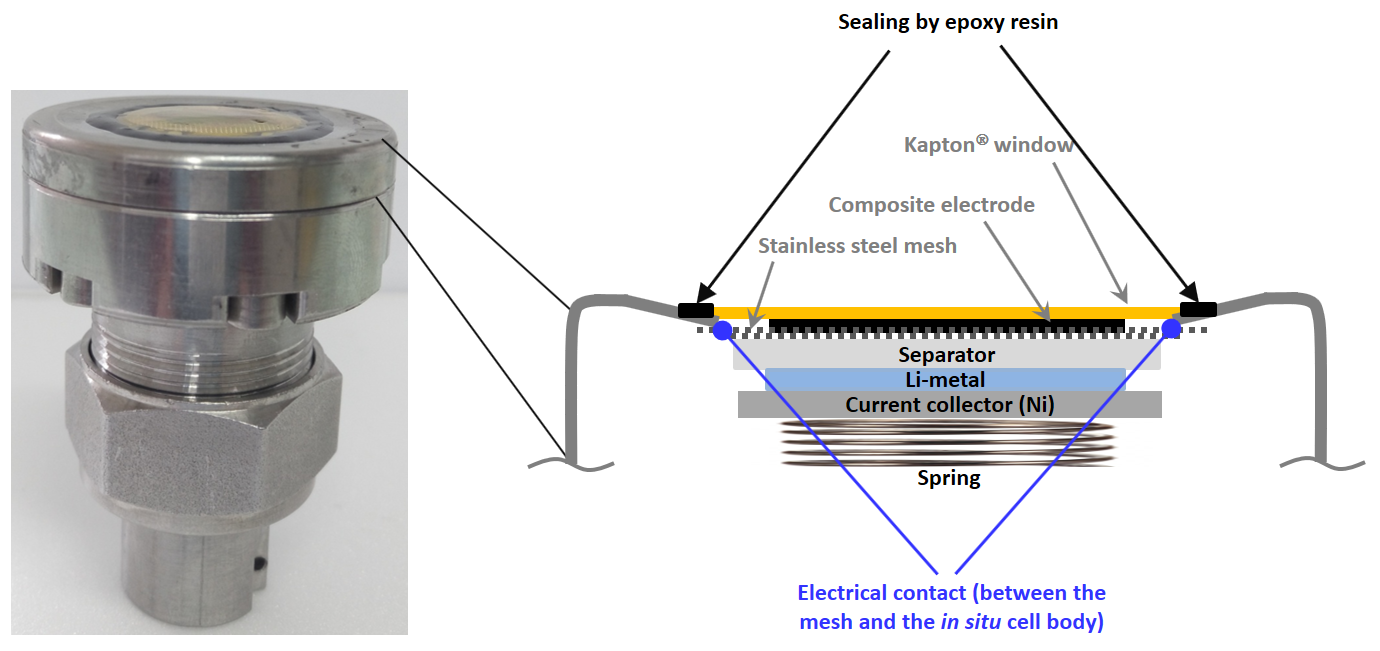


**Electron microscopy imaging and chemical element detection.** Scanning electron microscopy (SEM) measurements were collected on a JSM-7600F from JEOL. Transmission electron microscopy (TEM) imaging and selected-area electron diffraction (SAED) were performed on a Hitachi H-9000 NAR microscope (LaB_6_ source operated at 300 kV). The sample dispersed in diethylic ether was deposited onto a lacey carbon copper grid. Data were acquired on a Gatan Multiscan CCD camera. Elemental analysis, performed on a KEVEX Energy Dispersive X-ray Spectrometer (EDS) with Si(Li) diode, confirm a Mg/O ratio of about 6, close to the theoretical ratio.

**Chemicals.** 2,5-dihydroxyterephthalic acid H_4_-*p*-DHT (Sigma Aldrich, ≥ 98.0%), magnesium hydroxide extra pure Mg(OH)_2_ (Merck, 98.0%), lithium hydroxide monohydrate LiOH⋅H_2_O (Sigma Aldrich, ≥ 98.0%), lithium carbonate Li_2_CO_3_ (Sigma Aldrich, ≥ 98.0%), barium hydroxide octahydrate Ba(OH)_2_•8H_2_O (Prolabo, ≥ 99.0 %), calcium hydroxide anhydrous Ca(OH)_2_ (Sigma Aldrich, ≥ 98.0%), polyvininylidene fluoride PVDF (HSV 900) and *N*-methyl-2-pyrrolidone NMP (Sigma Aldrich, ≥ 99.0%). The common “LP30” battery grade electrolyte (i.e., LiPF_6_ 1 M in EC:DMC 1:1 vol./vol.) was directly employed as received from Novolyte. The employed conductive carbon was Ketjenblack EC-600JD (AkzoNobel), Super P (Timcal) and graphite nanoplatelets (GM5, XG Science).

**Synthesis of Mg(H_2_)-*p*-DHT(H_2_O)_5_⋅H_2_O.** To a heterogeneous solution of 2,5-dihydroxyterephthalic acid (5.2 g, 26.20 mmol, 1 eq) in ultra pure water (500 mL) was added Mg(OH)_2_ (1.53 g, 26.20 mmol, 1 eq). The solution was stirred at room temperature for 48 h. The obtained mixture was concentrated under reduced pressure to afford Mg(H_2_)-*p*-DHT(H_2_O)_5_⋅H_2_O (8.6 g, quant. yield); pale yellow crystals. IR (KBr pellet, cm^−1^) 3540 – 2970 (ν O-H)+(ν O-H)H_2_O, 1693-1625 (ν_as_ COO^−^), 1510-1450 (ν C=C), 1359 (ν_s_ COO^−^), 1267 (ν O-H /δ O-H) ), 875-794 (ν C-H); ^1^H NMR (400 MHz, D_2_O) δ 7.38 (s, 2H); ^13^C NMR (400 MHz, D_2_O) δ 174.34 (C, C=O), 151.37 (C, C-OH), 122.71 (C, C-COO^−^), 116.89 (C, C-H); Elemental analysis (calcd., found for Mg(H_2_)-*p*-DHT(H_2_O)_5_⋅H_2_O): C (29.25, 29.09), H (4.91, 4.73) wt.%; ICP-OES (calcd., found for Mg(H_2_)-*p*-DHT(H_2_O)_5_⋅H_2_O): Mg (7.40, 7.26) wt.%.

**Synthesis of Mg(Li_2_)-*p*-DHT⋅1.4 H_2_O**. To a degassed ultra-pure water (15 mL) were added successively in a glove box (MBRAUN, specially designed for working under Ar with moisture and containing less than 1 ppm of dioxygen), Mg(H_2_)-*p*-DHT)(H_2_O)_5_⋅H_2_O (600.0 mg, 1.83 mmol, 1 eq) and LiOH·H_2_O (153,3 mg, 3.7 mmol, 2 eq). The solution was stirred at room temperature for 16 h. The obtained mixture was dried under reduced pressure to afford an orange powder in quantitative yield. The water content was determined by thermogravimetry. IR (KBr pellet, cm^−1^) 3425 (ν O-H)H_2_O, 1602-1580 (ν_as_ COO^−^), 1476-1426 (ν C=C), 1367 (ν_s_ COO^−^), 1219 (ν CO−Li ), 890-804 (ν C-H); ^1^H NMR (400 MHz, D_2_O) δ 7.34 (2H, s); ^13^C NMR (400 MHz, D_2_O) δ 174.63 (C, C=O), 151.54 (C, C-O^−^), 123.12 (C, C-COO^−^), 117.08 (C, C-H); ICP-OES (calcd., found for Mg(Li_2_)-*p*-DHT⋅1.4H_2_O): Mg (9.37, 8.99), Li (5.35, 5.30) wt.%.

**Desolvation of Mg(Li_2_)-*p*-DHT⋅1.4 H_2_O**. Batches of 400 mg of powder (hand-grounded) were heated at 235°C in oven under air atmosphere over 48 hours. The resulting anhydrous Mg(Li_2_)-*p*-DHT compound was obtained in quantitative yield as khaki powder. IR (KBr pellet, cm^−1^) 1604 (ν_as_ COO^−^), 1476-1427 (ν C=C), 1367 (ν_s_ COO^−^), 1219 (ν CO-Li), 890-804 (ν C-H); ^1^H NMR (400 MHz, D_2_O) δ 7.34 (2H, s); ^13^C NMR (400 MHz, D_2_O) δ 174.6 (C, C=O), 151.52 (C, C-O^−^), 123.11 (C, C-COO^−^), 117.10 (C, C-H); ICP-OES (calcd., found for Mg(Li_2_)-*p*-DHT): Mg (10.46, 10.29), Li (5.98, 5.93) wt.%.

**Synthesis of Ba(Li_2_)-*p*-DHT**. To a degassed ultra-pure water (8 mL) were added successively in a glove box (MBRAUN, specially designed for working under Ar with moisture and containing less than 1 ppm of dioxygen), H_4_-*p*-DHT (400 mg, 2.02 mmol, 1 eq) and Ba(OH)_2_⋅8H_2_O (637.3 mg, 2.02 mmol, 1 eq). The solution was stirred at room temperature for 16 h, followed by 20 min at 50 °C: a suspension is obtained. To this precipitate was added LiOH⋅H_2_O (168.7 mg, 4.04 mmol, 2 eq). Again, the mixture was stirred at room temperature overnight. The pale green heterogeneous solution was dried under reduced pressure to afford a pale green powder. The as-prepared powder was then heated overnight at 130°C under vacuum in a Büchi B-585 glass oven Kugelrohr. The final anhydrous Ba(Li_2_)-*p*-DHT compound was obtained in quantitative yield as orange powder. The formation of Ba(Li_2_)-*p*-DHT was confirmed by NMR after a derivatization reaction based on a full reprotonation step ^2^: 4 drops of H_2_SO_4_ (concentrated) were added to a heterogeneous solution of Ba(Li_2_)-*p*-DHT (30 mg) in DMSO-d_6_ (1 mL). Both ^1^H and ^13^C liquid NMR spectra correspond to pure 2,5-dihydroxyterephthalic acid. ^1^H NMR (400 MHz, DMSO-d_6_ + H_2_SO_4_): δ 11.82 (s, H acid), 7.17 ppm (2H, s); ^13^C NMR (400 MHz, DMSO-d_6_ + H_2_SO_4_): δ 170.01 (C, C=O), 151.70 (C, C-OH), 119.33 (C, C-COOH), 117.26 (C, C-H); IR (KBr pellet, cm^−1^) 1558 (ν_as_ COO^−^), 1466-1417 (ν C=C), 1368 (ν_s_ COO^−^), 1223 (ν CO−Li), 872-804 (ν C−H); ICP-OES (calcd., found for Ba(Li_2_)-*p*-DHT): Ba (39.77, 39.48), Li (4.02, 4.13) wt.%.

**Synthesis of Ca(Li_2_)-*p*-DHT**. To a degassed ultra-pure water (8 mL) were added successively in a glove box (MBRAUN, specially designed for working under Ar with moisture and containing less than 1 ppm of dioxygen), H_4_-*p*-DHT (400 mg, 2.02 mmol, 1 eq) and Ca(OH)_2_ (149.7 mg, 2.02 mmol, 1 eq). The solution was stirred at room temperature for 16 h: a suspension is obtained. To this precipitate was added LiOH⋅H_2_O (168.7 mg, 4.04 mmol, 2 eq). Again, the mixture was stirred at room temperature overnight. The pale green heterogeneous solution was lyophilized to afford a pale orange powder. The as-prepared powder was then heated overnight at 200°C under vacuum in a Büchi B-585 glass oven Kugelrohr. The final anhydrous Ca(Li_2_)-*p*-DHT compound was obtained in quantitative yield as orange powder. The formation of Ca(Li_2_)-*p*-DHT was confirmed by NMR after a derivatization reaction based on a full reprotonation step ^2^: 4 drops of H_2_SO_4_ (concentrated) were added to a heterogeneous solution of Ca(Li_2_)-*p*-DHT (30 mg) in DMSO-d_6_ (1 mL). Both ^1^H and ^13^C liquid NMR spectra correspond to pure 2,5-dihydroxyterephthalic acid. ^1^H NMR (400 MHz, DMSO-d_6_ + H_2_SO_4_): δ 11.81 (s, H acid), 7.18 ppm (2H, s); ^13^C NMR (400 MHz, DMSO-d_6_ + H_2_SO_4_): δ 170.01 (C, C=O), 151.70 (C, C-OH), 119.33 (C, C-COOH), 117.26 (C, C-H); IR (KBr pellet, cm^−1^) 1591 (ν_as_ COO^−^), 1467-1413 (ν C=C), 1365 (ν_s_ COO^−^), 1215 (ν CO−Li), 876-811 (ν C−H); ICP-OES (calcd., found for Ca(Li_2_)-*p*-DHT): Ca (16.16, 15.58), Li (5.60, 5.13) wt.%.

**Synthesis of Li_2_(Li_2_)-*p*-DHT**. We first published the synthesis (and the electrochemical behavior) of this lithiated salt in 2013 ^2^ after having developed a green and innovative function-oriented synthesis method based on the chemical/biochemical CO_2_ sequestration^2^. However, for the sake of simplicity, we adopted in this new study the synthetic procedure reported by Chen’s group ^3^. Into a homogeneous solution of H_4_-*p*-DHT (1.0 g, 5.1 mmol, 1 eq) in anhydrous methanol (50 mL) was dripped 10.1 mL of lithium methoxide (2.2 M in methanol, 22.4 mmol, 4.4 eq). A yellow precipitate was formed after ~3 min under stirring at room temperature. After reaction for 40 h, the as-prepared solid was filtered and washed (3×6 mL) with anhydrous methanol, and dried under vacuum at 100°C overnight. The final compound Li_2_(Li_2_)-*p*-DHT was obtained by heating treatment at 220 °C for 24 h to afford Li_2_(Li_2_)-*p*-DHT as orange powder (yield: 87%). IR (KBr pellet, cm^−1^) 1570 (ν_as_ COO^−^), 1467-1412 (ν C=C), 1363 (ν_s_ COO^−^), 1232 (ν CO−Li ), 880-800 (ν C−H); ^1^H NMR (400 MHz, D_2_O) δ 7.11 (2H, s); ^13^C NMR (400 MHz, D_2_O) δ 175.97 (C, C=O), 152.78 (C, C-O^−^), 125.39 (C, C-COO^−^), 118.82 (C, C-H); ICP-OES (calcd., found for Li_2_ (Li_2_)-*p*-DHT): Li(12.51, 12.61) wt.%.

**Supplementary References**

1. Chen, H. *et al.* Lithium Salt of Tetrahydroxybenzoquinone: Toward the Development of a Sustainable Li-Ion Battery. *J. Am. Chem. Soc.* **131,** 8984–8988 (2009).

2. Renault, S. *et al.* A green Li–organic battery working as a fuel cell in case of emergency. *Energy Environ Sci.* **6,** 2124 (2013).

3. Wang, S. *et al.* Organic Li_4_C_8_H_2_O_6_ Nanosheets for Lithium-Ion Batteries. *Nano Lett.* **13,** 4404–4409 (2013).

4. Henkelis, S. E., McCormick, L. J., Cordes, D. B., Slawin, A. M. Z. & Morris, R. E. Synthesis and crystallographic characterisation of Mg(H_2_dhtp)(H_2_O)_5_·H_2_O. *Inorg. Chem. Commun.* **65,** 21–23 (2016).

5. Brown, I. D. What factors determine cation coordination numbers? *Acta Cryst. B* **44,** 545–553 (1988).

6. Morcrette, M. *et al.* In situ X-ray diffraction techniques as a powerful tool to study battery electrode materials. *Electrochim. Acta* **47,** 3137–3149 (2002).
